# Molecular docking analysis of PI3K/AKT with betulin from *Impatiens henslowiana*

**DOI:** 10.6026/973206300213269

**Published:** 2025-09-30

**Authors:** Rajamathanky Hariharan, Rajasekaran Aiyalu

**Affiliations:** 1 Department of Pharmacology, KMCH College of Pharmacy, Coimbatore, Tamil Nadu, India

**Keywords:** *Impatiens henslowiana*, *betulin*, *hepatoprotective*, *anti-inflammatory*, *antioxidant*, PI3K/AKT *signaling*

## Abstract

Drug-induced liver injury (DILI) remains a major clinical concern, as current therapies often show low efficacy and undesirable side effects. This study investigated the *hepatoprotective*, antioxidant, and anti-inflammatory potential of *Impatiens henslowiana* (IH) ethanolic extract and its active compound, betulin. IH was non-toxic up to 4000 mg/kg and showed protective effects in D-GalN-induced hepatotoxic rats at 200 and 400 mg/kg. Betulin exhibited strong antioxidant activity at a concentration of 9.5 µM and 19 µM in DCFH-DA assay, along with cytoprotective activity and downregulation of PI3K and AKT expression and their phosphorylation in HepG2 cells. Molecular docking confirmed high binding affinities of betulin to PI3K and AKT with binding energies of -10.36 kcal/mol and -10.99 kcal/mol, respectively, supporting its therapeutic potential.

## Background:

One of the serious adverse drug reactions is drug-induced liver injury (DILI) which in later stages leads to acute liver failure. According to Hosack *et al.*, (2023), nearly 14-19 patients in 100,000 people were found to be affected by acute liver injury. The two broad categories of DILI are intrinsic and idiosyncratic drug-induced liver injury -intrinsic DILI depends on the drug dose administered and its effect on the liver is identified within hours to days following drug exposure. Lipophilic drugs have the tendency to pass through the phospholipid bilayer of the liver cell, get converted into reactive metabolites by biomodification, leads to the onset of oxidative stress and initiation of various cellular signaling that leads to mitochondrial dysfunction and bile acid imbalance [1]. Idiosyncratic DILI cannot be predicted as the onset of liver injury occurs after weeks to months following drug administration (variable latency) and hence there lack of clarity in drug dosage [2]. Based on the liver injury pattern, idiosyncratic DILI were further subdivided into hepatocellular, cholestatic, or mixed depending upon the elevated liver enzyme levels (hypertransaminasemia) [3, 4]. Immune-mediated and non-immune-mediated DILI are further subdivisions of idiosyncratic DILI. Immune-mediated DILI occurs within 1-6 weeks after exposed to a drug and it is characterized by eosinophilia, rash, fever and production of autoantibodies. This further exacerbates to Stevens-Johnson syndrome [5]. Drug-induced autoimmune hepatitis was caused by nitrofurantoin and minocycline. On the other hand, when there is a delay in the onset of symptoms that varies from immune-mediated DILI results in non-immune-mediated DILI [5, 6]. DILI is characterized by the ratio of ALT and alkaline phosphatase equal to or greater than 5. This ratio is found to be equal to or less than 2 in patients with cholestatic DILI and in mixed DILI, the ratio lies in the range of 2-5 [7].

There are little information on the cause and clinical outcome of DILI in developing countries like India. The cases of tuberculosis are high in India and antituberculosis therapy was found to be the key factor for the onset of DILI [8]. Several inflammatory mediators have been identified to be involved in the progression of chronic liver disease, among which most of them were activators or targets of activators of nuclear factor-κB. Activation of NF-κB signaling in nonparenchymal cells will enhance inflammation, hepatocarcinogenesis and fibrosis [9]. On the other hand, inhibition of NF-κB affects the viability of hepatocytes. Hence, inhibition of the proteins in upstream of the signal transduction is required. PI3K/AKT signaling plays a key role in the activation of NF-κB [9]. Activation of PI3K/AKT signaling results in the phosphorylation of IκB, which in turn releases the NF-κB IH be complexed with NF-κB-IκB complex in the cytoplasm. Activated NFκB gets translocated to the nucleus where it binds to κB enhancer elements and induces the transcription of proinflammatory genes [10]. Currently, plant extracts and natural products have been widely used in the treatment of inflammatory disease. The phytochemicals present in plants hold promise as a good lead molecule that involves in rendering protection to hepatocytes from DILI. *Impatiens henslowiana* is a flowering plant that belongs to the family Balsaminaceae, is native to Sri Lanka and the Southern states of India, namely Tamil Nadu and Kerala [11]. Plants belonging to this family were used to treat various liver diseases, such as jaundice, due to their *hepatoprotective* activity [12]. Based on the above-described properties, the leaf extract of *Impatiens henslowiana* was investigated for its *hepatoprotective* activity in DILI. Among the existence of various phytochemicals in *Impatiens henslowiana*, betulin is found to be one of the active phytochemicals which is a pentacyclic triterpene and found to exhibit various activities such as anticancer, antiviral, antibacterial and antifungal [13]. This terpene is prevalent in plants belonging to the Betulaceae family. Betulin is found to exhibit anti-inflammatory activity by inhibiting the COX-2 enzymes in macrophages [14]. Therefore, it is of particular interest to investigate the *hepatoprotective* potential of *Impatiens henslowiana* leaf extract against drug-induced liver injury.

## Materials and Methods:

## Reagents:

Most of the reagents were purchased from Sigma Aldrich. These reagents included ethanol (99%), chloroform, ascorbic acid, Dulbecco's Modified Eagle Medium (DMEM), Fetal bovine serum (FBS), Silymarin, D- galactosamine, 3-(4,5-dihydrothiazol-2-yl)-2,5-diphenyltetrazolium bromide (MTT), Dimethyl Sulfoxide (DMSO) and sodium carboxymethylcellulose.

## Cell lines:

HepG2 (Human liver hepatocellular carcinoma) cell lines obtained from the National Centre for Cell Science (NCCS) in Pune, India were utilized to perform cell-based assays. Cells were grown in DMEM that were supplemented with 10% fetal bovine serum and kept in a humified atmosphere with 5% CO2 at 37°C. The stock cultures were maintained in 25 cm^2^ culture flasks.

## Animals for *in vivo* experiments:

*In vivo* studies were performed using Male Wistar rats (250 - 300g) were obtained from Sri Venkateshwara Enterprises, Bangalore. The animals were grown under normal laboratory conditions at a temperature of 25 ± 3°C, 12h light/dark cycle with a humidity of 50 ± 5% for two weeks. Standard pellet diet and water ad libitum were provided to the rats. Ethical approval to carry out the experiment was obtained from the institutional animal ethical committee of KMCH College of Pharmacy, Coimbatore, Tamil Nadu (India) No. IAEC/ Reg.No.685 /PO/Re/S/2002 /CPCSEA. IAEC No. KMCRET /Ph.D /01/2019-20.

## Acute toxicity study:

The whole plant of *Impatiens henslowiana* was air-dried and grinded into fine powder. About 500g of the powdered sample was taken and subjected to solvent extraction in a Soxhlet apparatus using ethanol. The filtrate was then dried in a rotary evaporator at 40°C. The obtained concentrated extract was stored at 4°C for further use. An acute toxicity study was performed based on the limit test procedure according to the guidelines of the Organization for Economic Co-operation and Development, OECD-425 (2000). Five groups of rats were assigned randomly, and each group was provided with different doses (250, 500, 1000, 2000, and 4000 mg/kg) of IH via the oral route and any behavioral changes were monitored for the next 24 and 72h for a total of 14 days [15]. No toxicity or mortality was seen even up to the dose of 2000 mg/kg. Based on the result, 200 mg/kg and 400 mg/kg were selected to investigate the *hepatoprotective* activity of the extract.

## D-Galactosamine-induced hepatotoxicity studies:

A total of 30 male Wistar rats were divided into five groups and each group consisted of 6 animals:

[1] Group 1: Treated with 1mL/kg of 0.3% sodium carboxymethylcellulose orally for 21 days and served as the normal control.

[2] Group 2: Treated with 400mg/kg of D-Galactosamine via an intraperitoneal route on the 21st day, which serves as the negative control.

[3] Group 3: 200mg/kg of IH was administered orally along with 400mg/kg of D-Galactosamine given by intraperitoneal route for 21 days.

[4] Group 4: 400mg/kg of IH was administered orally along with 400mg/kg of D-Galactosamine given by intraperitoneal route for 21 days.

[5] Group 5: Intraperitoneal injection of 400 mg/kg of D-Galactosamine, along with oral administration of a standard drug, silymarin (100 mg/kg) for 21 days, serves as a positive control group.

After 21 days, the treated rats were anesthetized by administering 500mg/kg of ketamine via the intraperitoneal route. Blood samples were collected to perform biochemical analyses. Liver tissue was removed, cleaned with ice-cold phosphate-buffered saline, and kept at -20 °C for histopathological studies.

## Assessment of serum biochemical parameters:

The levels of alanine aminotransferase (ALT), aspartate aminotransferase (AST), total protein, alkaline phosphatase (ALP), gamma-glutamyl transferase (γ-GT), and total bilirubin in all the treated and untreated rats were determined to predict the effect of IH, d-galactosamine, and Silymarin.

## Assessment of *in vivo* Antioxidant activity:

The antioxidant activity of the IH on liver tissue was evaluated as per the method performed by Shaikh *et al.* [16]. The excised liver was weighed and homogenized in 0.1M cold phosphate buffer (pH 7.4). The homogenate was then centrifuged at 10,000 rpm for 10 mins at 4°C to collect post-mitochondrial supernatant (PMS). Again, the homogenate was centrifuged at 17,000 rpm for an hour. The concentration of the antioxidant scavengers namely superoxide dismutase (SOD), catalase (CAT) and reduced glutathione (GSH) in the liver homogenate was evaluated.

## Histopathological analysis:

The liver tissue of the treated and untreated rats was removed after sacrificing it and washed with saline. To carry out histopathological analysis, the liver tissue was fixed with neutral buffered formalin. 5 µm thickness of liver tissue was prepared using a rotary microtome and a paraffin-embedded block was made. Following this, the tissue was stained with hematoxylin and eosin, and the histoarchitecture of the liver was viewed using an Olympus DP 72 microscope (Tokyo, Japan) [17].

## Isolation of active constituents from *Impatiens henslowiana*:

Dried powder of *Impatiens henslowiana* was dissolved in methanol and adsorbed onto a 60-120 microns silica gel. This was then loaded into a silica gel column (mesh size 100-200 microns), which was packed with hexane solvent. Elution was carried out with an increase in the polarity of the hexane and ethyl acetate combination (90:10; 80:20; 50:50; 40:60; 20:80; 10:90), where the final elution phases comprised ethyl acetate. Subsequent elution was performed with ethyl acetate and chloroform combined in various ratios (90:10; 80:20; 60:40; 50:50; 40:60; 30:70; 20:80; 10:90) where the final elution consisted of 100% chloroform. Final elution was performed with chloroform and methanol at different ratios (98:2; 96:4; 94:6, 93:7; 92:8; 90:10; 85:15; 80:20). A total of 98 fractions was obtained and TLC was performed with these fractions. TLC result shows that fractions 16 to 24 show a single spot with a retention factor (Rf) of 0.62. Based on the results, these fractions were consolidated and the solvent present in it was removed under pressure. The crude solid was then treated with activated charcoal in hot ethanol and placed at room temperature for crystallization. A colorless solid was obtained which is characterized using UV-Vis spectroscopy, FT-IR spectroscopy, 1H-NMR, 13C-NMR and Mass spectrometry.

## Spectral characterization of betulin by UV-visible spectroscopy, FT-IR, H1-NMR, C13-NMR and Mass spectrometry:

The isolated compound was dissolved in ethanol and its absorbance was measured at the wavelength between 200 and 800 nm using a JASCO UV-Vis spectrophotometer. The analysis of the functional groups was determined using a PerkinElmer FT-IR spectrometer. This is performed by the potassium bromide (KBr) pellet (FTIR grade) method and the characteristic bands were found at the frequency range from 650 to 4000 cm^-1^. FTIR is carried out through the potassium bromide (KBr) pellet (FTIR grade) method and the distinctive bands are seen within the frequency range from 650 to 4000 cm^-1^. In addition, 1H and 13C NMR spectra of the compound were determined with a Bruker NMR spectrometer (Agilent DD2 300 MHz), where the compound is dissolved in CDCl_3_ and measured at 300 and 75 MHz, respectively. The chemical shifts are given as D-values and it is reported in parts per million (ppm) with tetramethylsilane (TMS, d = 0) as internal standard. Mass spectroscopic characterization of the isolated compound was carried out in MicrOTOF-Q III (Bruker Daltonik GmbH, Germany) in the electron spray (ESI) negative mode, with a capillary voltage of 4000 V, nebulizer pressure: 2.0 bar; drying gas: 8 L/min at 300°C, where the range of mass-to-charge ratio was set between 50 to 1,000 m/z.

## *In vitro* cytotoxic activity of Betulin:

The cytotoxic activity of betulin in the HepG2 cell line was evaluated using an MTT reduction assay (Kupcsik, 2011). HepG2 cells were treated with 2.5, 5, 10, 20 and 40µM concentrations of betulin and incubated for 24 hours. Untreated cells serve as a control. After incubation, the cells were washed with 200 µL of PBS and then 25 µL of MTT in PBS was added and kept undisturbed for 3 hours. Then the dye is discarded and 150 µL of DMSO is added which dissolves the formed formazan crystals in the cells. The viability of the cell is calculated by measuring the absorbance at 570 nm using an ELISA microplate reader. A graph is plotted with the concentration of betulin along the x-axis and the cytotoxicity percentage along the y-axis. From the graph, the IC50 value of betulin is obtained.

## DCFH-DA staining:

The antioxidant activity of betulin was determined by using 2',7'-dichlorodihydrofluorescein diacetate (DCFH-DA) staining [18] in HepG2 cell lines. The cells were initially incubated with 10 µM concentration of D-galactosamine for 24 hours. At the end of incubation, 9.5 and 19 µM of betulin were added to the cells and allowed to stand for 3 hours. Cells treated only with D-galactosamine serve as a negative control, whereas untreated cells serve as a control. After incubation, the cells were washed with PBS buffer and 25µM of DCFH-DA dye was added and allowed to stand for 30 mins. The dye was discarded at the end of incubation and washed with PBS and the intensity of fluorescence was measured in fluorescence microscopy.

## Western blotting:

Western blot analysis was performed to evaluate the expression blockade of PI3K, AKT, pPI3K and pAKT treated with betulin. HepG2 cells were incubated with 9.5 and 19 µM of betulin for a period of 72 hours. After incubation, the cells were lysed and the lysate was loaded in a 7% polyacrylamide gel to carry out electrophoresis. After electrophoresis, the proteins were transferred onto a nitrocellulose membrane. Blocking with non-fat dry milk for 1 hour and then the membranes were incubated with the antibodies, that is, monoclonal antibodies directed for PI3K, AKT, pPI3K and pAKT, overnight at 4 °C. Secondary antibodies, namely horseradish peroxidase-conjugated goat-anti-mouse IgG and goat-anti-rabbit IgG (both: dilution 1:1000, Dako, Denmark) were added and incubated at room temperature for 30 minutes. The membranes were then treated with ECL detection reagent and the protein bands were visualized with a Sapphire Imager. The ratio of protein intensity and whole protein intensity was calculated and given in percentage and is related to the controls that have not been treated with any drugs.

## Molecular docking:

A molecular docking study of betulin against the PI3K and AKT proteins was performed in the Autodock 4.2 tool [19]. The crystal structure of the PI3K protein complexed with an inhibitor LY294002 and AKT protein crystallized with an indazole-pyridine inhibitor was obtained from Protein Data Bank (PDB ID-1E7V) [20] and (PDB ID- 2UZT) [21]. Co-crystallized ligands and water present in the 3D structure of these two proteins were removed with the help of Pymol software [22]. The 2D structure of the betulin was obtained from PubChem [23] and is transformed to a PDB file with Open Babel software [24]. The energy minimization of betulin was done by making use of the steepest descent algorithm and chemical force field in the Avogadro software [25]. The optimized proteins and betulin were used to perform docking studies in AutoDock 4.2 software. Partial Kollman charges and polar hydrogen atoms were added to the proteins and then saved as "pdbqt" file, which has the solvation parameter coordinates. Torsion angles and hydrogen atoms were assigned to betulin and are saved as "pdbqt" format. The binding site of PI3K and AKT proteins was obtained from the literature [20-21]. The grid box determines the region where betulin is allowed to bind with the target protein and it is generated by using 40 x 40 x 40 points and a grid spacing of 0.375 Å. The co-crystalized inhibitor at the active site of these proteins was removed and betulin was allowed to bind at that region. 100 GA runs were carried out by using the Lamarckian genetic algorithm. The docking results of the PI3K-betulin complex and AKT-betulin complex were obtained from the top clusters based on inhibition concentration, binding energy (kcal/mol), hydrogen bond interaction and least root mean square deviation (RMSD).

## Results and Discussion:

The acute toxicity of IH was determined by providing different doses (250, 500, 1000, 2000, and 4000 mg/kg) via the oral route to Wistar male rats. No mortality was seen in any of the treated rats which confirms that IH is devoid of toxicological effects. In addition, notable signs of toxicity such as constipation, eye color change, aggression, and other behavioral changes were not seen for 14 days in any of the rats following the administration of IH. The *in vivo* *hepatoprotective* effect of IH towards d-galactosamine-induced hepatotoxicity was determined in Wistar male rats. Serum biochemical parameters, which include AST, ALT, total bilirubin, total protein, gamma-glutamyl transferase and ALP levels were determined to investigate liver damage and dysfunction. The results for the various experimental groups are summarized in Table 1 (see PDF), which shows the comparison of biochemical parameters across different treatments.

This study rigorously evaluated the *hepatoprotective* efficacy of IH extract through systematic *in vivo* experiments across five distinct rat groups subjected to hepatotoxicity and therapeutic interventions. Group 1 served as the normal control, receiving sodium carboxy methylcellulose, which has all biochemical parameters within normal ranges (AST: 52.07±3.72 IU/L; ALT: 35.37±2.12 IU/L; ALP: 43.63±5.41 IU/L; GGT: 3.52±0.26 IU/L; total bilirubin: 0.40±0.06 mg/dL; total protein: 8.53±0.52 g/dL), confirming the baseline health of the liver. Group 2, the negative control, was treated with 400 mg/kg of d-galactosamine, leading to significantly elevated levels of liver enzymes and bilirubin (AST: 188.53±5.98 IU/L; ALT: 98.41±5.63 IU/L; ALP: 177.20±6.60 IU/L; GGT: 16.18±1.18 IU/L; total bilirubin: 1.81±0.17 mg/dL) and a marked reduction in total protein (2.73±0.37 g/dL), indicative of severe hepatotoxicity. Conversely, Group 3, receiving a low dose of IH (200 mg/kg) alongside d-galactosamine, demonstrated substantial protective effects with notable reductions in enzyme levels and bilirubin (AST: 125.67±4.82 IU/L; ALT: 61.12±3.56 IU/L; ALP: 88.80±8.07 IU/L; GGT: 8.32±0.45 IU/L; total bilirubin: 0.82±0.05 mg/dL) and an increase in total protein (6.27±0.19 g/dL) compared to Group 2. This suggests that IH at this dosage effectively mitigates liver damage to a considerable extent.

Group 4, administered a high dose of IH (400 mg/kg) and exhibited even more pronounced *hepatoprotective* effects, closely paralleling the efficacy of the standard drug, silymarin. This group saw significant reductions across all parameters measured (AST: 89.93±6.98 IU/L; ALT: 52.70±2.91 IU/L; ALP: 80.73±7.17 IU/L; GGT: 5.75±0.33 IU/L; total bilirubin: 0.71±0.10 mg/dL) and an elevation in total protein (6.58±0.27 g/dL), reinforcing IH's strong potential in liver damage amelioration. Finally, Group 5, the standard control, treated with silymarin (100 mg/kg) and d-galactosamine, demonstrated near-complete reversal of liver damage, showcasing the expected therapeutic benefits of silymarin with liver enzyme and bilirubin levels nearly returning to normal (AST: 61.41±5.59 IU/L; ALT: 36.90±3.28 IU/L; ALP: 48.90±3.90 IU/L; GGT: 4.92±0.49 IU/L; total bilirubin: 0.43±0.05 mg/dL; total protein: 6.73±0.14 g/dL). These comprehensive findings, depicted in Figures 1-6 (see PDF), underscore the potent *hepatoprotective* and antioxidant properties of *Impatiens henslowiana* extract, particularly when administered at higher dosages, offering significant protection against chemically induced liver injury.

## *In vivo* Antioxidant potential of IH:

The antioxidant potential of IH in d-galactosamine-induced hepatotoxicity in male Wistar rats is determined by measuring the level of oxidative stress scavengers in the liver tissues. The levels of the oxidative stress scavengers namely glutathione (GSH), glutathione peroxidase (GPx), catalase (CAT) and superoxide dismutase (SOD) in the liver tissue homogenate of the IH-treated and untreated rats after 24 hrs of the last dose are measured and the levels are given in Table 2 (see PDF). The graphical representation of the level of antioxidant scavengers was given in Figure 7-10 (see PDF). Rats in the untreated group exhibited high levels of hepatic antioxidants with GSH (39.06 ± 4.53 U/mg), GPx (38.97 ± 1.14 U/mg), SOD (7.42 ± 0.42 U/mg), and CAT (49.38 ± 1.22 U/mg), which indicates that the hepatocytes are free from oxidative stress. On the contrary, rats treated with d-galactosamine showed a decrease in the levels of GSH (2.73 ± 0.37 U/mg), GPx (2.60 ± 0.29 U/mg), SOD (4.50 ± 0.30 U/mg), and CAT (3.00 ± 0.20 U/mg), which indicates the oxidative stress induced by d-galactosamine leading to liver damage. Silymarin, the standard drug used against oxidative stress, was found to have the following levels: GSH (42.80 ± 0.83 U/mg), GPx (46.05 ± 1.38 U/mg), SOD (8.79 ± 0.78 U/mg), and CAT (54.27 ± 1.28 U/mg), which are almost similar to those of the normal rats. Rats treated with d-galactosamine and low- and high-dose (200 and 400 mg/kg) IH were found to have an increase in the levels of oxidative scavengers. In the low-dose treated group, the concentration was found to be GSH (39.06 ± 4.53 U/mg), GPx (38.97 ± 1.14 U/mg), SOD (7.42 ± 0.42 U/mg), and CAT (49.38 ± 1.22 U/mg), which is greater than that of the d-galactosamine-only treated group. This suggests that the free radicals produced due to d-galactosamine were scavenged by IH through the increased production of antioxidant scavengers. In the same way, at a high dose of 400 mg/kg, the levels of GSH (42.80 ± 0.83 U/mg), GPx (46.05 ± 1.38 U/mg), SOD (8.79 ± 0.78 U/mg), and CAT (54.27 ± 1.28 U/mg) were almost similar to that of the silymarin-treated group. These results confirm the radical scavenging activity of IH.

## Histopathological analysis:

Based on the results from the biochemical parameters of liver tissue of IH-treated rats, the *hepatoprotective* of the IH was further confirmed by histopathological studies. The histopathology of rats in Group I exhibits a normal arrangement of hepatocytes and it is given in Figure 11a (see PDF). Inflammation, hemorrhage and coagulative necrosis were seen in group II rats which is due to the oxidative stress caused by d-galactosamine (Figure 11b - see PDF) leading to liver damage and liver toxicity. Figure 11c (see PDF) shows an improvement with moderate inflammation and mild tissue necrosis in the liver tissue from the rats being treated with a low dose of 200mg/kg of IH. At a higher dose of IH at 400mg/kg, the liver section has mild necrosis when compared to group 2 (Figure 11d - see PDF). This confirms that IH conserves the histoarchitecture and exhibits *hepatoprotective* activity. In group V where the liver tissue appears to be normal due to the treatment with a standard drug, silymarin (Figure 11e - see PDF).

[1] Group I: normal arrangement of hepatocytes.

[2] Group II: section of liver tissue of D-galactosamine-treated group showing massive coagulative necrosis, hemorrhage, and inflammation.

[3] Group III: section of liver tissue treated with 200 mg/kg IH and D-galactosamine, showing mild inflammation.

[4] Group IV: section of liver tissue treated with 400 mg/kg IH and D-galactosamine, showing normal histology with mild inflammation.

[5] Group V: section of 100 mg/kg Silymarin liver tissue pretreated in the liver followed by D-galactosamine, showing preservation of normal hepatocytes (40x magnification).

## UV-Visible Spectral analysis:

The maximum absorbance (λmax) of the isolated phytochemical was found at 209 and 264 nm (figure 12 - see PDF) which confirms the presence of aromatic ring and conjugation within the phytochemical. The presence of an aromatic ring with extensive conjugation suggests that the isolated compound is a triterpenoid.

## FT IR examination:

The FTIR chromatogram of IH is given in figure 13 (see PDF). The spectrum of the isolated compound gave characteristic peaks of groups present in the isolated compound like 3401 cm^-1^ (-OH group); 2941 cm^-1^ and 2868 (-CH stretch), 1642 cm^-1^ C=C; 1454 cm^-1^ (CH2); 1029 (C-O). The peak at 3401 cm^-1^ refers to the -OH which indicates the presence of hydroxyl group.

## 1H and 13C NMR investigation:

1H-NMR (DMSO-d6): δ ppm: 4.64 - 4.65 (d,1H,H-29); 4.52 (t,1H,H-28); 4.24 - 4.18 (m,2H,H3,H-28); 4.01 (s,1H,H-3); 3.47 - 3.54 (m,1H,H-2); 3.06 - 3.07(m,1H, H-2); 2.94 - 2.96 (m,1H,H-19); 2.62 - 2.65 (m,1H,H-13); 2.32 - 2.39 (m,1H,H-14); 1.82 - 1.88 (m,2H, H-21,H-22); 1.60 - 1.65 (m,4H,H-1,H-12,H-13, H-15,); 1.52 - 1.58 (m,2H,H-6,H-18); 1.40 - 1.49 (m,3H,H-11,H-16,H4); 1.33 - 1.35 (m,4H,H-7); 1.22 - 1.28 (m,2H,H-9); 0.80 - 0.98 (m,18H, H-5,H-23,H-26,H-27); 0.77 (s,3H,H-25); 0.64 (s,3H,H-24). Based on the Proton NMR presence of 50 Protons has been identified indicating the presence of a triterpenoid type of compound (Figure 14 - see PDF) [26].

13C-NMR (DMSO-d6): δ ppm: 150.32 (C-20). 109.55(C-29), 76.73 (C3), 57.88 (C28), 54.81 (C5), 49.79 (C9), 48.12 (C18), 47.34(C17), 47.27(C19), 42.16(C14), 40.41(C8), 40.12(C4), 38.45(C1), 38.20(C17), 36.63 (C10), 33.77 (C7), 29.26(C16), 28.98(C21), 28.05(C23), 27.11(C2), 26.62(C15), 24.78(C12), 20.31(C11), 18.71(C30), 17.92(C6), 15.85(C25), 15.75(C24), 15.64(C26), 14.47 (C27) Totally 30 signals are observed between 150 to 14.47ppm indicates that 30 carbon atoms are present in the compound (Figure 15 - see PDF).

## Mass spectroscopy:

The isolated compound's mass spectrum showed a noticeable molecular ion peak at m/z 443.4 (M+1) (Figure 16 - see PDF). This is in good agreement with the compound's estimated molecular weight of 442 g/mol, which is determined using the chemical formula C30H50O2. The existence of an extra hydrogen atom is indicated by the measured mass's slight increase above the expected value, which might be the result of a small impurity or an isotopic variety. When a molecule loses one electron, a radical cation with a charge of +1 is created. This ion is represented by the molecular ion peak (M+) in mass spectra. In this instance, the molecular ion peak at m/z 443.4indicates that the molecular weight of the chemical is 442 g/mol, which is in line with the molecular formula. From this spectral characterization, the isolated compound was identified as betulin (Figure 17 - see PDF) [26].

## MTT assay of betulin:

The most common and simplest assay used to determine the cell viability is the MTT assay. This assay is based on the conversion of 3-[4, 5-dIHthylthiazol-2-yl]-2, 5-diphenyltetrazolium bromide (MTT) to formazan crystals by the mitochondrial enzyme in live cells. The cytoprotective function of betulin was analyzed by MTT assay where the HepG2 cells were incubated with 2.5, 5, 10, 20 and 40 µM and later the cell viability was calculated. Figure 18 (see PDF) shows the cell density with an increase in the concentration of betulin. There is a decrease in the count of viable cells in a dose-dependent manner of betulin (Figure 19 - see PDF). From the figure, the IC50 of betulin was found to be 23.97 µM. The cell viability based on the concentration of betulin is given in table 3 (see PDF). This shows the cell membrane permeability property of betulin. Thus, based on these analyses the dose below the IC50 was considered to be cytoprotective and used for further *hepatoprotective* activity studies.

## Antioxidant activity of betulin:

DCFH-DA staining was used to evaluate the antioxidant activity of betulin. In this assay, DCFH-DA is converted to DCFH by the esterase found in the cytoplasm of the cell and later undergoes oxidation by the free radicals, namely ROS to form 2'-7'dichlorofluorescein (DCF), which emits fluorescence. The intensity of fluorescence indicates the oxidative stress within the cell and this is measured using fluorescence microscopy. Figure 20 (see PDF) shows the fluorescence intensity of the betulin, d-galactosamine and untreated cells. From the figure, it is clear that the fluorescence intensity is higher in d-galactosamine due to the oxidative stress and the rise in the free radicals was decreased in betulin-treated cells which results in a decrease in the fluorescence intensity. Thus, it is evident that betulin has the ability to neutralize the free radicals in the cells, which confirms its antioxidant activity [27].

## Effects of Betulin on PI3K/AKT signaling:

Activation of the PI3K/AKT signaling pathway results in the activation of various other pathways such as LPS, TNF-α-mediated pro-inflammatory, NF-κB and apoptotic pathways. This is achieved by the phosphorylation of IκB which leads to enhanced cytokine secretion and inflammatory reactions. Thus, the current study involves in the inhibition of the PI3K and AKT which prevents the onset of other downstream signaling. Phosphorylated PI3K and AKT proteins indicate the activation of PI3K/AKT signaling pathway. The protein expression levels of PI3K, AKT, pPI3K and pAKT in treated and untreated cells were determined using Western blotting. It has been found that levels of PI3K, AKT, pPI3K and pAKT were upregulated in cells treated with d-galactosamine which results in the onset of inflammation in liver cells. On the other hand, the levels of PI3K, AKT, pPI3K and pAKT were downregulated in cells treated with 9.5 and 19 µM of betulin. The expression level of these proteins is given in figure 21A-H (see PDF).

## Molecular docking:

From the protein expression analysis, it is evident that the levels of PI3K, AKT, pPI3K and pAKT are upregulated in d-galactosamine, results in the onset of inflammation in the hepatocyte due to its hepatotoxic activity. Hence, inhibition of PI3K and AKT proteins by betulin was studied through molecular docking (Figure 22 - see PDF). Docking with PI3K shows that betulin binds with a low binding energy of -10.4 kcal/mol with an inhibition constant of 21.4nm (Table 4 - see PDF). The 3D and 2D interaction of the Betulin-PI3K complex was given in Figure 23 (see PDF). The hydroxyl group of betulin forms a hydrogen bond with the VAL882 and ASP950 at a bond distance of 3.1 and 2.6 Å. This complex is stabilized by various weak interactions such as hydrophobic interaction, van der Waals, etc. THR347, GLU398, GLU406, LYS432, TYR433, ILE438, SER441, GLY442 and ASN447 in the active site of PI3K render hydrophobic interaction with betulin. HIS405 is involved in pi-pi interaction with betulin [20]. Betulin binds with AKT protein at a low binding energy of -10.99 kcal/mol and exhibits a low inhibition constant of 8.85nM (Table 5 - see PDF). The 3D and 2D interaction of Betulin-AKT complex was given in Figure 24 (see PDF). Betulin-AKT complex was stabilized by hydrogen bonds and other weak interactions with the key amino acids residues at the active site. The hydroxyl group of betulin hydrogen bond with oxygen of ARG19 at a bond distance of 3.4 Å, and with the nitrogen of LYS168 at a distance of 3.0 Å. Weak interactions that stabilizes the complex is conferred by ASN20, ALA21, LEU49, GLY50, THR51, GLY52, VAL57, LYS72, MET120, TYR122, GLU127, GLU170, ASN171, THR183, ASP184, PHE327 and TYR330 [21].

## Conclusion:

The anti-inflammatory, antioxidative and *hepatoprotective* activity of IH was due to the presence of betulin in it. Data shows the importance of betulin as an effective agent in the protection of the liver against drug-induced liver injury. These findings not only reinforce the therapeutic potential of betulin but also underscore its promise as a lead candidate for the development of *hepatoprotective* agents.

## Abbreviations:

AKT - Protein kinase B

CV - Central vein

IκB -IkappaB kinase

H - Hepatocytes

IH - *Impatiens henslowiana* ethanol extract

KC - Kupffer cells

NF-κB - Nuclear factor kappa-B

PI3K - Phosphoinositide 3-kinase

ROS - Reactive Oxygen Species

S - Sinusoids

## Author agreement statement:

We, the undersigned declare that this manuscript is original, has not been published before and is not currently being considered for publication elsewhere. We confirm that the manuscript has been read and approved by all named authors and that there are no other persons who satisfied the criteria for authorship but are not listed. We further confirm that the order of authors listed in the manuscript has been approved by all of us. We understand that the Corresponding Author is the sole contact for the Editorial process. He/she is responsible for communicating with the other authors about progress, submissions of revisions and final approval of proofs.

## Declaration of preprint status:

The authors confirm that no part of this manuscript has been previously posted to any preprint server, nor are there preprint versions of this work available online.

## Declarations:

## Funding:

No funding was received for conducting this study.

## Ethical approval:

Not applicable.

## Consent for publication:

Not applicable.

## Informed consent:

Not applicable.

## Clinical trial number:

Not applicable.
